# Arabidopsis Ovate Family Proteins, a Novel Transcriptional Repressor Family, Control Multiple Aspects of Plant Growth and Development

**DOI:** 10.1371/journal.pone.0023896

**Published:** 2011-08-23

**Authors:** Shucai Wang, Ying Chang, Jianjun Guo, Qingning Zeng, Brian E. Ellis, Jin-Gui Chen

**Affiliations:** 1 Key Laboratory of Molecular Epigenetics of MOE and Institute of Genetics & Cytology, Northeast Normal University, Changchun, China; 2 Department of Botany, Northeast Agricultural University, Haerbin, China; 3 Department of Botany, University of British Columbia, Vancouver, British Columbia, Canada; 4 Michael Smith Laboratories, University of British Columbia, Vancouver, British Columbia, Canada; 5 Biosciences Division, Oak Ridge National Laboratory, Oak Ridge, Tennessee, United States of America; University of South Florida College of Medicine, United States of America

## Abstract

**Background:**

The Arabidopsis genome contains 18 genes that are predicted to encode Ovate Family Proteins (AtOFPs), a protein family characterized by a conserved OVATE domain, an approximately 70-amino acid domain that was originally found in tomato OVATE protein. Among AtOFP family members, AtOFP1 has been shown to suppress cell elongation, in part, by suppressing the expression of *AtGA20ox1*, AtOFP4 has been shown to regulate secondary cell wall formation by interact with KNOTTED1-LIKE HOMEODOMAIN PROTEIN 7 (KNAT7), and AtOFP5 has been shown to regulate the activity of a BEL1-LIKEHOMEODOMAIN 1(BLH1)-KNAT3 complex during early embryo sac development, but little is known about the function of other AtOFPs.

**Methodology/Principal Findings:**

We demonstrated here that AtOFP proteins could function as effective transcriptional repressors in the Arabidopsis protoplast transient expression system. The analysis of loss-of-function alleles of *AtOFPs* suggested *AtOFP* genes may have overlapping function in regulating plant growth and development, because none of the single mutants identified, including T-DNA insertion mutants in *AtOFP1*, *AtOFP4*, *AtOFP8*, *AtOFP10*, *AtOFP15* and *AtOFP16*, displayed any apparent morphological defects. Further, *Atofp1 Atofp4* and *Atofp15 Atofp16* double mutants still did not differ significantly from wild-type. On the other hand, plants overexpressing *AtOFP* genes displayed a number of abnormal phenotypes, which could be categorized into three distinct classes, suggesting that *AtOFP* genes may also have diverse functions in regulating plant growth and development. Further analysis suggested that AtOFP1 regulates cotyledon development in a postembryonic manner, and global transcript profiling revealed that it suppress the expression of many other genes.

**Conclusions/Significance:**

Our results showed that AtOFPs function as transcriptional repressors and they regulate multiple aspects of plant growth and development. These results provided the first overview of a previously unknown transcriptional repressor family, and revealed their possible roles in plant growth and development.

## Introduction

*OVATE* gene was originally identified as a major quantitative trait locus that controls fruit shape in tomato. A single mutation in *OVATE* led to a premature stop codon can cause the transition of tomato fruit from round- to pear-shaped [1]. OVATE gene encodes a protein with a putative nuclear localization signal and an approximately 70-aa C-terminal domain that is conserved in tomato, Arabidopsis, and rice [1]. This 70-aa C-terminal conserved domain was also known as the Domain of Unknown Function 623 (DUF623) that is found exclusively in plant proteins. Subsequently, this domain was designated as OVATE domain, and proteins contain this domain were designated as Ovate Family Proteins (OFPs) [2].

OFP proteins are plant-specific family of regulatory proteins [2]. In Arabidopsis, there are 18 genes that are predicted to encode proteins containing OVATE domain, and most members of this family also contain a predicted nuclear localization signal but lack recognizable DNA binding domains [2], [3]. Nine of these 18 proteins were found to interact with 3-amino acid loop extension homeodomain (TALE) proteins in a yeast two-hybrid screen [2], suggesting that AtOFP proteins are components of TALE homeodomain protein network.

So far, only a few AtOFPs have been shown to regulate plant growth and development [3], [4], [5]. AtOFP1 was shown to function as a transcriptional repressor and has a role in regulating cell elongation, in part, by suppressing the expression of *AtGA20ox1*, a gene encoding the key enzyme in gibberellin (GA) biosynthesis [3]. AtOFP5 was reported to negatively regulate the activity of a BLH1-KNAT3 complex during early embryo sac development [4]. More recently, we showed that AtOFP4 participated in the regulation of secondary cell wall formation by interacting with KNAT7 [5].

To further explore the function of AtOFPs in plant growth and development, we took a combination of bioinformatic, molecular, biochemical, and genetic approaches to analyze *AtOFP* genes in Arabidopsis. We provide evidence that all AtOFP proteins tested could function as transcriptional repressors in Arabidopsis protoplast transient expression system, and that *AtOFP* genes likely have overlapping and diverse function in regulating plant growth and development. By using a dexamethasone (DEX) inducible system, we also showed that AtOFP1 regulates cotyledon development in a postembryonic manner, and identified a list of genes whose expression is suppresses by AtOFP1.

## Results

### Ovate Family Proteins in Arabidopsis

**Figure 1:**
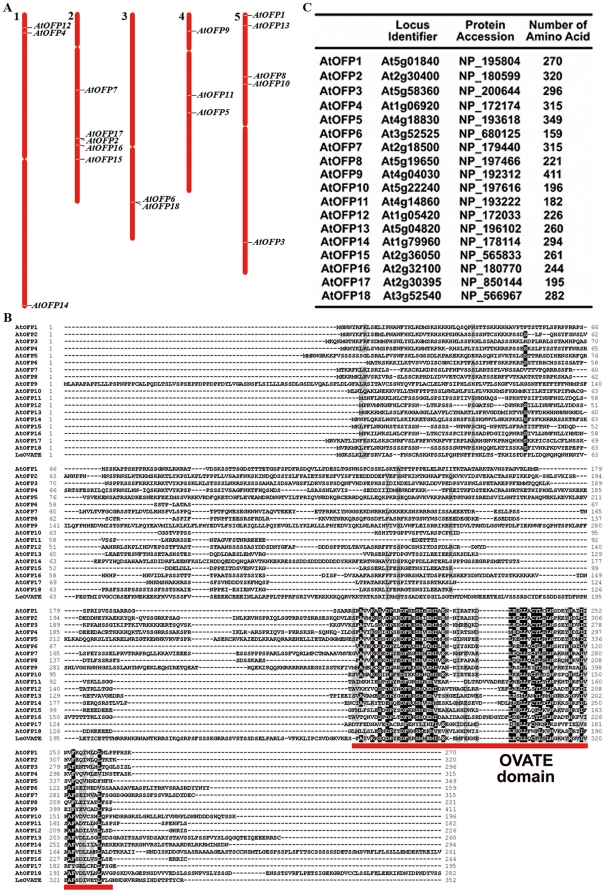
AtOFP proteins family. (**A**) Genome distribution of *AtOFP* genes. (**B**) Amino acid sequence alignment of the AtOFP proteins. Identical amino acids are shaded in black, and similar amino acids are shaded in grey. Red under line indicates the OVATE domains. The sequence alignment was generated by the ClustalW multiple alignment of BioEdit Sequence Alignment Editor (http://www.mbio.ncsu.edu/BioEdit/bioedit.html). (**C**) Arabidopsis locus identifiers, protein accession numbers, and length of predicted amino acids of AtOFPs.

There are 18 genes in the Arabidopsis genome that are predicted to encode OVATE domain-containing proteins [2], [5]. These 18 genes distributed in all five chromosomes of the Arabidopsis genome (Figure 1A). The OVATE domain is mostly present in the C-terminus of these proteins (Figure 1B). The majority of OFP proteins are 200–350 amino acids in length (Figure 1C). Amino acid sequence alignment indicated that other regions of these proteins tend to have lower sequence identity/similarity, and that AtOFP proteins share sequence similarity largely in the OVATE domain (Figure 1B).

### AtOFP Proteins Function as Transcriptional Repressors

**Figure 2:**
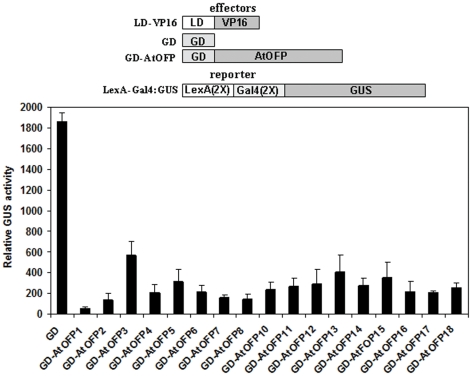
AtOFPs are transcriptional repressors. Effector gene, transactivator and reporter gene were cotransfected into protoplasts derived from Arabidopsis rosette leaves. GUS activity was assayed after 20–22 h incubation in darkness. Effectors and reporter constructs were diagrammed at the top of the figure.

**Figure 3:**
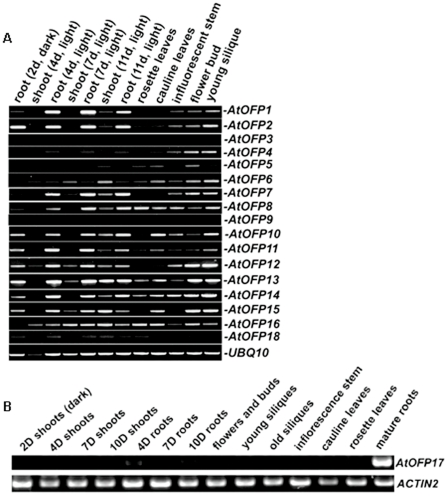
Expression of *AtOFP* genes in various tissues and organs of Arabidopsis. The expression of *UBIQUITIN10 (UBQ10)* or *ACTIN2* was used as a control.

Previously, we have shown that AtOFP1 is a transcriptional repressor that suppresses cell elongation [3]. Plants overexpressing AtOFP2, 4 and 7 have similar phenotype as plant overexpressing AtOFP1, including kidney-shaped cotyledons and round and curled leaves, and we showed that AtOFP2, 4 and 7 also act as transcriptional repressors [3], [5]. These results prompted us to further test if all other AtOFP proteins can also function as transcriptional repressors. To do that, the same Arabidopsis protoplast transient expression system used to assess the transcriptional activity of AtOFP1, 2, 4, 7 was used [3], [5]. As shown in Figure 2, all AtOFP proteins tested could function as transcriptional repressors, indicating that AtOFPs may represent a novel transcriptional repressor family. Because *AtOFP9* transcript is undetectable (Figure 3), and *AtOFP9* gene contains a large intron, we failed to make the *35S:GD-AtOFP9* construct. As a result, AtOFP9 was not included in this assay.

### Expression of *AtOFP* Genes

To characterize AtOFPs' function in plants, we examined the expression pattern of all *AtOFP* genes across various tissues and organs (Figure 3). We have detected the transcript of all the *AtOFP* genes except *AtOFP9*. It should also be mentioned that all *AtOFP* genes, except *AtOFP9* and *AtOFP17*, are intronless (data not shown). Comparison among the expression patterns of *AtOFP* genes revealed that several *AtOFPs*, such as *AtOFP1, AtOFP2,* and *AtOFP7*, had largely overlapping expression pattern among various tissues and organs with relatively stronger expression in roots and floral organs, and several other *AtOFPs*, such as *AtOFP6*, *AtOFP12*, and *AtOFP16*, showed ubiquitous expression in all tissues/organs examined (Figure 3A). In our initial search of OVATE domain-containing proteins in Arabidopsis, we did not find AtOFP17. After Hackbusch et al. [2] reported their findings, we included AtOFP17 in our analysis. We found that AtOFP17 did appear to be an OVATE domain-containing protein, though it had relatively lower sequence similarity with other AtOFP proteins at the OVATE domain (Figure 1B) and did not pair with any other AtOFP proteins in the phylogenetic analysis [5]. Because the original RNA samples prepared for analyzing the expression patterns of other *AtOFP* genes were no longer available, we prepared a new set of RNA samples to analyze the expression pattern of *AtOFP17*. We found that *AtOFP17* was highly expressed in roots (Figure 3B). These results, together with the results that all AtOFPs tested function as transcriptional repressors, suggested that AtOFPs may have both overlapping and non-overlapping functions in regulating plant growth and development.

### AtOFPs Regulate Plant Growth and Development

**Figure 4:**
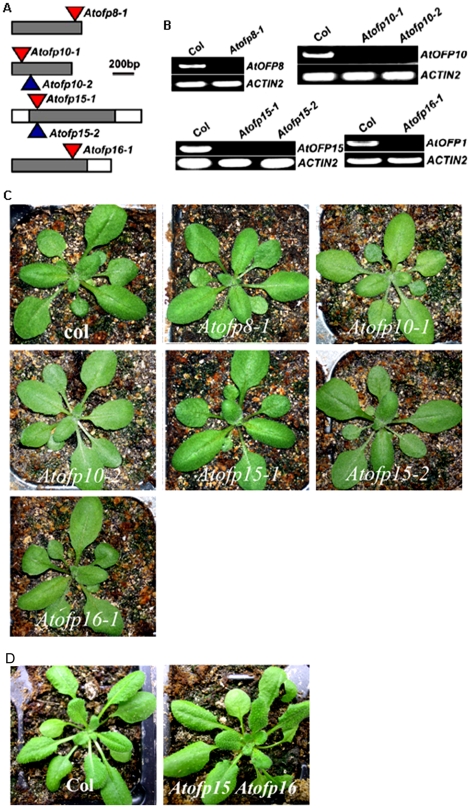
T-DNA/transposon insertional alleles of AtOFPs. (**A**) A diagram to illustrate the T-DNA or transposon insertion site in the *Atofp* mutants. Gray boxes represent exons. The T-DNA and transposon inserts are not drawn to scale. (**B**) RT-PCR analysis of *AtOFP* transcript in corresponding *Atofp* mutants. RNA was isolated from 7d-old light-grown seedlings. *AtOFP* gene-specific primers were used to amplify the full-length *AtOFP* transcript. The expression of *ACTIN2* was used as a control. (**C**) *Atofp* single mutant plants have wild-type morphology. (**D**) *Atofp15-1Atofp16-1* double mutant has wild-type morphology. Note that pictures for *Atofp15-1Atofp16-1* double mutant and its wild-type control in (**D**) were not taken at the same time with other genotypes in (**C**).

We previously analyzed putative loss-of-function alleles of *AtOFP1* and *AtOFP4* [3], [5]. Both *Atofp1-1* and *Atofp4-2* mutants had wild-type morphology, although detailed analysis showed that cell wall thickness is affected in *Atofp4-2* mutants [3], [5]. We interpreted that *AtOFP1* and *AtOFP4* may have overlapping function with other members of *AtOFP* gene family. In order to further address this, we sought all available T-DNA or transposon insertional alleles of all other *AtOFP* genes. We have isolated insertional mutants in *AtOFP8*, *AtOFP10*, *AtOFP15*, and *AtOFP16* (Figure 4A). RT-PCR analyses indicated that corresponding full-length *AtOFP* transcript was absent in each mutant allele (Figure 4B), implying that they likely represent loss-of-function alleles of these genes. However, like *Atofp1-1* and *Atofp4-2* mutant, none of these mutants displayed any apparent morphological defects (Figure 4C).

Having failed to identify any significant morphological defects in loss-of-function alleles of *AtOFP* genes, we turned our attention to gain-of-function approach. We reasoned that overexpression of redundant AtOFP genes would confer similar phenotypes. Previously, we have shown that plants overexpressing *AtOFP2*, *AtOFP4* or *AtOFP7* had similar phenotypes with plants overexpressing *AtOFP1* [3], [5]. We wanted to further test if overexpression of any other *AtOFP* genes would also phenocopy plants overexpressing *AtOFP1*. Therefore, we generated transgenic plants overexpressing each of all other AtOFPs except AtOFP9, fused in-frame with an N-terminal HA tag (HA-AtOFP) in the wild-type Col background. Since we could not detected transcript for *AtOFP9* in all tissues and organs tested, a full length genomic fragment of *AtFOP9* was used instead of its CDS, and the *AtOFP9* construct does not contain an N-terminal HA tag. The expression of each *AtOFP* was driven by the *35S* promoter.

**Figure 5:**
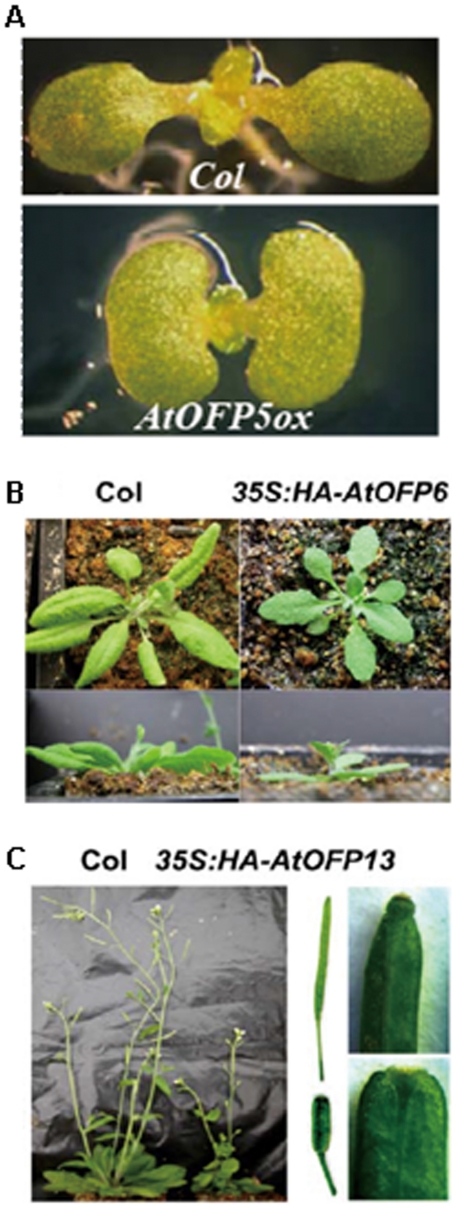
Phenotypes of plants overexpressing *AtOFPs*. (**A**) Class I phenotypes that were observed in plants overexpressing *AtOFP1*, *AtOFP2*, *AtOFP4*, *AtOFP5*, or *AtOFP7*. Shown are 7-day old, light-grown wild-type Col (top) and a plant overexpressing *HA-AtOFP5* (bottom). (**B**) Class II phenotypes that were observed in plants overexpressing *AtOFP6* or *AtOFP8*. Shown on top are top views of five-week-old Col (left) and a plant overexpressing *HA-AtOFP6* (right). Shown on bottom are side views of Col (left) and *35S:HA-AtOFP6* plant. (**C**) Class III phenotypes that were observed in plants overexpressing *AtOFP13*, *AtOFP15*, *AtOFP16*, or *AtOFP18*. Shown in left are seven-week-old Col (left) and a plant overexpressing *HA-AtOFP13* (right). Shown in the middle are the siliques of Col (top) and *35S:HA-AtOFP13* plants (bottom). Shown in right are close views of the siliques of Col (top) and *35S:HA-AtOFP13* (bottom).

Analysis of plants overexpressing each individual *AtOFP* gene revealed several interesting observations. First, in addition to *AtOFP2*, *AtOFP4* and *AtOFP7*, plants overexpressing *AtOFP5* also phenocopied plants overexpressing *AtOFP1* (Figure 5A, Table 1). The characteristic phenotypes of these plants were kidney-shaped cotyledons and round and curled leaves. Therefore, we designated *AtOFP1*, *AtOFP2*, *AtOFP4*, *AtOFP5* and *AtOFP7* as Class I *AtOFP* genes. Second, plants overexpressing *AtOFP6* and *AtOFP8* displayed very unique phenotypes. The characteristic phenotypes of these plants were flat, thick and cyan rosette leaves (Figure 5B, Table 1). We designated *AtOFP6* and *AtOFP8* as Class II *AtOFP* genes. Third, plants overexpressing *AtOFP13*, *AtOFP15*, *AtOFP16* and *AtOFP18* have similar phenotypes and were distinct from plants overexpressing other *AtOFPs* (Figure 5C, Table 1). These plants had blunt-ended siliques. Therefore, we designated *AtOFP13*, *AtOFP15*, *AtOFP16* and *AtOFP18* as Class III *AtOFP* genes. Finally, we have yet to observe any obvious morphological phenotypes of plants overexpressing other *AtOFPs* including *AtOFP3*, *AtOFP9*, *AtOFP10*, *AtOFP11*, *AtOFP12*, *AtOFP14* and *AtOFP17* (Table 1).

**Table 1:** Phenotypes of plants overexpressing AtOFPs.

	AtOFPs	Phenotypes
Class I	AtOFP1, AtOFP2, AtOFP4, AtOFP5, AtOFP7	Kidney-shaped cotyledons, round and curled leaves, small rosette size, later flowering, reduced fertilization and round seeds.
Class II	AtOFP6, AtOFP8	Flat, thick and cyan leaves, enhanced apical dormancy
Class III	AtOFP13, AtOFP15, AtOFP16, AtOFP18	Small rosette size, later flowering, reduced fertilization, and blunt-end siliques.
Others AtOFPs		No visible phenotypes observed

Since all the loss-of-function mutants obtained so far for AtOFPs, including double mutant *Atofp1 Atofp4*, do not confer any apparent morphological defects (Figure 4C) [3], [5], while over-expression of some AtOFPs confer similar phenotypes (Figure 5, Table 1), it was tempting to speculate that AtOFPs function redundantly to regulate plant growth and development. To further address the potential functional redundancy among AtOFP genes, we have generated *Atofp15 Atofp16* double mutant, because AtOFP15 and AtOFP16 were both grouped in Class III. As shown in Figure 4D, *Atofp15 Atofp16* double mutant is indistinguishable from wild-type plants, supporting the view of functional redundancy among these genes.

### AtOFP1 Regulates Cotyledon Development in a Postembryonic Manner

**Figure 6:**
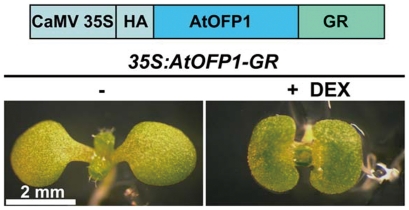
AtOFP1 controls cotyledon development in a postembryonic manner. Cotyledon phenotypes of *35S:AtOFP1-GR* seedlings in the absence (left) and presence (right) of 10 µM DEX. Shown are 6d-old, light-grown seedlings. A schematic diagram of The DEX-inducible system used in this study was diagrammed on the top of the figure.

To get further insights into the action of AtOFPs in the regulation of plant growth and development, we took AtOFPs-regulated cotyledon development as an example because cotyledon development is initiated during embryogenesis and plants overexpressing *AtOFP1*, *AtOFP2*, *AtOFP4*, *AtOFP5* and *AtOFP7* have kidney-shaped cotyledons. However, it was unclear whether this characteristic phenotype was caused during embryogenesis or at the postembryonic level. In order to address this question, we utilized the dexamethasone (DEX)-inducible expression system in which AtOFP1 was fused with the glucocorticoid receptor (GR) (*35S:AtOFP1-GR*) (Figure 6). AtOFP1 was chosen among the 5 AtOFPs that could cause kidney-shaped cotyledons was because AtOFP1 has been shown to work as a transcriptional repressor to regulate plant growth and development [3]. We found that without DEX treatment, the cotyledons of *35S:AtOFP1-GR* line had wild-type morphology (Figure 6). When seeds of *35S:AtOFP1-GR* line were sown on MS/G medium containing DEX, remarkably, *35S:AtOFP1-GR* seedlings produced characteristic kidney-shaped cotyledons (Figure 6), phenocopying *AtOFP1ox* seedlings. These findings further confirmed that AtOFP1 functions in the nucleus, consistent with its role as an transcriptional repressor, and it also demonstrated that the characteristic kidney-shaped cotyledons observed in *AtOFP1ox* seedlings is due to the postembryonic action of *AtOFP1*.

### AtOFP1 Suppresses Gene Expression in Young Seedling

Having showed that AtOFP1 functions in the nucleus to regulate cotyledons development in a postembryonic manner by using the DEX inducible system, we wanted to further look for genes whose expression is controlled by AtOFP1 using this system. DEX inducible system has been well used to examine the target genes of many other transcription factors. For example, by taking this approach, it has been demonstrated that *Ntc12*, a tobacco *GA20-oxidase* gene is a direct target gene of NTH15, a tobacco KNOX homeodomain protein [6].

**Figure 7:**
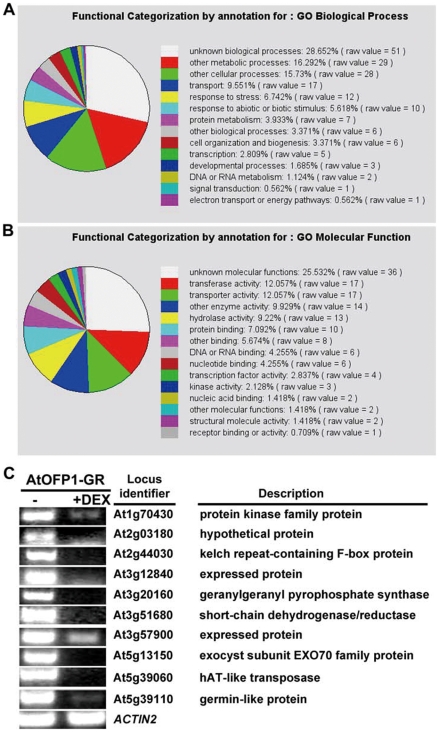
AtOFP1 suppresses gene expression. Functional categorization was based on a total of 129 genes whose expression was down-regulated at least 2.0-fold in *35S:AtOFP1-GR* with DEX treatment over without DEX treatment. (**A**) Functional categorization by annotation for GO biological process. (**B**) Functional categorized by annotation for GO molecular function. (**C**) Validation of microarray data by RT-PCR. Ten genes whose expression is down-regulated at least 2.0-fold by AtOFP1 in microarray analysis were selected and examined by RT-PCR analysis. PCR was performed with 30 cycles. The expression of *ACTIN2* was used as a control.

In order to discover candidate target genes for AtOFP1 in a global manner, we conducted microarray analysis using *35S:AtOFP1-GR* line. Total RNAs were isolated from young light-grown seedlings with or without DEX treatment. We used the GeneChip Arabidopsis ATH1 Genome Arrays. Each array contains more than 22,500 probe sets representing approximately 24,000 genes. Transcriptomic comparisons were performed to discover genes that were differentially expressed in the presence of DEX over in the absence of DEX. We specifically looked for genes whose expression was down-regulated by DEX treatment. We found that a total of 129 genes showed at least 2-fold down-regulation by DEX treatment (Table S1). Remarkably, about one-fourth of these AtOFP1-down-regulated genes act in unknown biological processes or have unknown molecular functions (Figure 7A, B). Proteins encoded by other genes are categorized to function as transferases (∼12%), transporters (∼12%), hydrolases (∼9%) and other enzymes (∼10%). We performed RT-PCR to validate the microarray data. We examined the expression of 10 selected genes in *35S:AtOFP1-GR* young seedlings with DEX treatment, compared with no DEX treatment. We found that consistent with the microarray data, the transcript of these 10 genes was clearly down-regulated in the DEX-treated *35S:AtOFP1-GR* young seedlings (Figure 7C).

## Discussion

OVATE domain-containing proteins are found exclusively in plants. The Arabidopsis genome encodes 18 proteins that are predicted to contain OVATE domain. Rice and poplar genomes also encode over 10 such proteins respectively. However, little is known about the function of these proteins. Here we provided a combination of bioinformatic, biochemical, molecular, and genetic evidence that Arabidopsis OVATE family proteins could function as effective transcriptional repressors, and that *AtOFP* genes may work in a redundant manner to regulate plant growth and development.

### AtOFPs Represent a Novel Plant Specific Transcriptional Repressor Family With Redundant Functions

Transcription factors control almost all aspects of plant growth and development. In Arabidopsis, there are more than 5% genes that have been identified to encode transcription factors. The identification of AtOFP1 as an active transcriptional repressor indicates that AtOFPs might represent an unidentified transcriptional repressor family. Indeed, all AtOFP proteins tested could function as effective transcriptional repressors in the Arabidopsis protoplast transient expression system (Figure 2).

Loss-of-function study has been a straightforward strategy and a useful approach to assign functions to a particular gene. To study the function of AtOFPs in plant, we have isolated putative loss-of-function alleles of *AtOFP1*, *AtOFP4*, *AtOFP8*, *AtOFP10*, *AtOFP15*, and *AtOFP16* (Figure 4) [3], [5]. However, none of these single mutants displayed any apparent morphological defects (Figure 4C). Further, *Atofp1 Atofp4* and *Atofp15 Atofp16* double mutants still had wild-type morphology (Figure 4D)[5]. These results suggest that there is functional redundancy among *AtOFP* genes. Similar situation happened with the study of other transcription factor gene family. For example, *Aux/IAA* genes encode transcription factors that are critical regulators controlling auxin-regulated gene expression [7], [8], [9], [10], [11]. There are 29 *Aux/IAA* genes in Arabidopsis genome. None of the loss-of-function single mutants displayed any apparent morphological defects [12]. A combination of loss-of-function mutations in multiple *Aux/IAA* genes still did not yield obvious morphological phenotypes [12], suggesting that there is possibly high functional redundancy among *Aux/IAA* genes.

Our results indicated that the high functional redundancy may exist among AtOFP genes, though a higher order combination of mutations in closely related AtOFP genes is required to address this further. However, loss-of-function alleles of *AtOFP2*, *AtOFP5* and *AtOFP7* which are in the same Class I as *AtOFP1* and *AtOFP4* (Table 1) have yet to be available. Similarly, loss-of-function alleles of *AtOFP13* and *AtOFP18* which are in the same Class III as *AtOFP15* and *AtOFP16* are also unavailable at the present time. We have attempted to use other approaches to silence closely related *AtOFP* genes. However, *AtOFP* genes are different from each other at the nucleotide level, which made it an inefficient approach (data not shown). Nevertheless, our initial analyses of *Atofp* single and double mutants and over-expression studies suggest that *AtOFP* genes may act in a redundant manner.

### AtOFPs Regulate Multiple Aspects of Plant Growth and Development

Although loss-of-function studies did not reveal any significant information about the molecular function of *AtOFPs,* gain-of-function studies by ovexexpressing *AtOFP* genes have provided some hints about their possible roles in plant development. We have expressed each of 18 *AtOFP* genes in Arabidopsis. Plants overexpressing *AtOFPs* displayed a number of interesting phenotypes (Figure 5, Table 1). These phenotypes can be categorized into three classes: the Class I (*AtOFP1*, *AtOFP2*, *AtOFP4*, *AtOFP5* and *AtOFP7*) phenotypes are kidney-shaped cotyledons with round and curled leaves; the Class II (*AtOFP6* and *AtOFP8*) phenotypes are flat, thick and cyan leaves; and the Class III (*AtOFP13*, *AtOFP15*, *AtOFP16* and *AtOFP18*) phenotypes are blunt-ended siliques. Other *AtOFP* genes (*AtOFP3*, *AtOFP9*, *AtOFP10*, *AtOFP11*, *AtOFP12*, *AtOFP14* and *AtOFP17*) were uncategorized because we have yet to observe any obvious phenotypes of plants over-expressing these genes. Interestingly, we noticed that such classification is largely consistent with the results of phylogenetic analysis [5] and expression pattern analysis (Figure 3).

We are cautious that overexpression study may not reveal the authentic function of these proteins, and should be interpreted together with other studies. In the lack of any significant phenotypes of loss-of-function alleles of *Atofp* single and double mutants, results from overexpression studies may provide some clues about their possible roles in plant development. For examples, the phenotype of blunt-ended siliques observed in plants overexpressing *AtOFP13*, *AtOFP15*, *AtOFP16* or *AtOFP18* were also observed in *erecta* mutants and heterotrimeric G-protein β subunit mutant, *agb1* [13], [14], [15].

Another possible approach to study the function of AtOFP proteins is through site-directed mutagenesis or TILLING [16]. Such approach has proven to be very useful for studying the function of Aux/IAA proteins [9], [10]. On the basis of amino acid sequence alignment of the OVATE domain of all AtOFP proteins, we noticed that a few amino acids within the OVATE domain are completely conserved among all AtOFP proteins (Figure 1B). These amino acids are therefore potential targets for site-directed mutagenesis or screening for TILLING mutations for future studies.

### AtOFP1 Regulates Cotyledon Development and Gene Expression

Cotyledons are considered to be a model system to study leaf development [17]. Overexpression of *AtOFP2*, *AtOFP4*, *AtOFP5* or *AtOFP7* conferred similar cotyledon phenotypes as overexpression of *AtOFP1* (Figure 5, Table 1), suggesting that class I *AtOFP* genes may have overlapping function in regulating cotyledon development. By using the DEX-inducible expression system, we showed that AtOFP1 regulates cotyledon development in a postembryonic manner (Figure 6). Apparently, the function of class I AtOFPs is not restricted to cotyledon development, because alternations in the shape of leaves and floral organs have also been observed in *AtOFP1ox* and *AtOFP4ox* lines [3], [5].

Previously we showed that AtOFP1 is localized in the nucleus and suppresses the expression of *AtGA20ox1* [3]. Here we show that AtOFP1 also functions in the nucleus to regulate cotyledon development (Figure 6), and suppresses, directly or indirectly, the expression of many other genes (Figure 7, Table S1). About one-third of proteins encoded by AtOFP1-down-regulated genes are annotated to function as transferases, transporters or hydrolases. One of the most remarkable observations was that about one-fourth of the AtOFP1-down-regulated genes are predicted to encode protein with unknown molecular functions (Figure 7, Table S1). It would be interesting to investigate the function of these genes in cotyledon development.

In summary, we provided evidence that AtOFP proteins may represent a novel transcriptional repressor family and that they function redundantly to regulate multiple aspects of plant growth and development. By using a DEX inducible system, we showed that AtOPF1 regulate cotyledon development in a postembryonic manner and that AtOFP1 suppresses the expression of many other genes.

## Materials and Methods

### Plant Materials and Growth Conditions

All mutants, transgenic lines, and wild-type are in Arabidopsis Columbia ecotypic background (Col). All mutants were obtained through the Arabidopsis Biological Resources Center (Columbus, Ohio). For plant grown in soil, seeds were germinated and grown in 2×2 inch pots containing moistened 1∶3 mixture of Sunshine Mix #1 (Sun Gro Horticulture Canada Ltd., Seba beach, Alberta, Canada) and Metro-Mix 220 (W.R. Grace & Co. of Canada, Ontario, Canada) with 14/10 hr photoperiod at approximately 120 µmol m^−2^ s^−1^ at 22°C. For seedlings used for phenotypic analysis and RT-PCR analysis, seeds were surface sterilized and grown on 0.6% (w/v) phytoagar (plantmedia, Dublin, Ohio) solidified ½ Murashige & Skoog (MS) basal medium with vitamins (plantmedia, Dublin, Ohio) and 1% (w/v) sucrose.

### Isolation of T-DNA/Transposon Insertional Mutants of *AtOFPs*

A transposon insertion mutant allele of *AtOFP1*, *Atofp1-1*, and a T-DNA insertion mutant allele of *AtOFP4*, *Atofp4-2* has been reported previously [3], [5]. All other mutants of *AtOFP* genes were identified through searching the SALK T-DNA Express database (http://signal.salk.edu/cgi-bin/tdnaexpress) [18] and the Exon Trapping Insert Consortium (EXOTIC) database (http://www.jic.bbsrc.ac.uk/science/cdb/exotic/index.htm), and obtained through the Arabidopsis Biological Resources Center (Columbus, Ohio). A T-DNA insertion mutant allele of *AtOFP8*, SALK_049190, was designated as *Atofp8-1*. T-DNA insertion mutant alleles of *AtOFP10*, SAIL_1231_D07 and SAIL_406_B07, were designated as *Atofp10-1* and *Atofp10-2*, respectively. Transposon insertional mutant alleles of *AtOFP15*, SM_3_40468 and SM_3_19375, were designated as *Atofp15-1* and *Atofp15-2*, respectively. A transposon insertional mutant allele of *AtOFP16*, SM_3_3082, was designated as *Atofp16-1*. *AtOFP* gene-specific primer and the T-DNA specific primer JMLB1 (5′-GGCAATCAGCTGTTGCCCGTCTCACTGGTG-3′) or the transposon element-specific primer (5′-TACGAATAAGAGCGTCCATTTTAGAGTGA-3′) were used for PCR genotyping. The presence/absence of the full-length *AtOFP* transcript in each insertion line was examined by RT-PCR.

### Generation of Double Mutants

The *Atofp1 Atofp4* double mutant has been reported previously [5]. The *Atofp15-1 Atofp16-1* double mutants were generated by crossing *Atofp15-1* into *Atofp16-1*. For simplicity, the *Atofp15 Atofp16* double mutant nomenclature refers to the *Atofp15-1 Atofp16-1* double mutant.

### Plasmid Construction and Plant Transformation

To generate the *35S:HA-AtOFP* and *35S:GD-AtOFP* constructs, the full-length open-reading frame (ORF) of each AtOFP gene was amplified by PCR using genomic DNA isolated from 10-day old, light-grown Arabidopsis seedlings, because all *AtOFP* genes, except *AtOFP9* and *AtOFP17*, contain a single exon. The PCR fragment was then cloned in frame with an amino terminal HA or GD tag into the *pUC19* vector under the control of the double *35S* enhancer promoter of *CaMV* [19.20]. To generate *35S:HA-AtOFP1-GR* construct, GR was fused with AtOFP1 in *35S:HA-AtOFP1* construct. Corresponding constructs with HA tag in *pUC19* vector were digested with a restriction enzyme, *Eco*R I, then sub-cloned into binary vector *pPZP211* or *pPZP221* for plant transformation [21]. *35S:GD-AtOFP*constructs in *pUC19* vector were used for plasmid DNA isolation and protoplasts transfection.

Wild-type Col-0 plants were used to transform with related constructs in *Agrobacterium tumefaciens* GV3101 by the floral dip method [22]. Phenotypes of transgenic plants were examined in the T1 generation, and confirmed in T2 up to T4 generations. For all transgenic plants, at least 5 transgenic lines with similar phenotypes were obtained.

### Protoplasts Isolation, Transfection and GUS Activity Assay

The procedures of protoplast isolation, transfection and GUS activity assays have been described previously [3], [19], [20]. GUS activities were measured by using a Fluoroskan Finstruments Microplate Reader (MTX Lab Systems, Inc., Vienna, Virginia, USA).

### RNA Isolation and RT-PCR

Total RNA was isolated from seedlings or different tissues/organs of grown-up plants using the TRIzol reagent (Invitrogen Canada Inc., Burlington, Ontario, Canada). cDNA was synthesized using 1 µg total RNA by Oligo(dT)-primed reverse transcription, using OMNISCRIPT RT Kit (QIAGEN). *ACTIN2* (*ACT2*) (amplified by primers 5′-CCAGAAGGATGCATATGTTGGTGA-3′ and 5′-GAGGAGCCTCGGTAAGAAGA-3′) or *UBIQUITIN10* (*UBQ10*) (amplified by primers 5′-GATCTTTGCCGGAAAACAATTGGAGGATGGT-3′ and 5′-CGACTTGTCATTAGAAAGAAAGAGATAACAGG-3′) were used as controls in PCR reactions.

### Microarray Analysis

*35S:AtOFP1-GR* seeds were directly sown on MS/G plates without or with 10 µM DEX and stratified at 4°C in dark for 2 days. Imbibed seeds were then transferred to growth conditions (22°C with 14/10 hr photoperiod at approximately 120 µmol m^−2^ s^−1^) and grown for 7d. Total RNA was isolated, purified and concentrated using the RNeasy Plant Mini Kit (QIAGEN, Mississauga, Ontario, Canada). The GeneChip Arabidopsis ATH1 Genome Arrays (http://www.affymetrix.com/support/technical/datasheets/arab_datasheet.pdf) were used. Each array contains more than 22,500 probe sets representing approximately 24,000 genes. cDNA synthesis, cRNA labeling, fragmentation, hybridization, washing and scanning were performed according to the procedures suggested by the manufacturer (GeneChip Expression Analysis Technical Manual, http://www.affymetrix.com/support/downloads/manuals/expression_analysis_technical_manual.pdf) and conducted at the Wine Research Centre DNA Array platform at University of British Columbia (http://www.landfood.ubc.ca/wine/micro/microarray.html). The transcript levels of the whole genome genes were classified into three categories by detection p-values: present (p<0.04), marginal (0.04<p<0.06), and absent (p>0.06). Functional categorization of genes was done through the TAIR Gene Ontology (GO) Annotations (http://www.arabidopsis.org/tools/bulk/go/index.jsp).

All data is MIAME compliant and that the raw data for one of the two replicates has been deposited in the MIAME compliant database EBI, with accession numbers E-MEXP-3222.

## Supporting Information

Table S1 List of genes whose expression is suppressed at least 2.0 fold by AtOFP1.
(DOC)
