# Supplementary material for: Arabidopsis Ovate Family Proteins, a Novel Transcriptional Repressor Family, Control Multiple Aspects of Plant Growth and Development
Source: PLoS One. 2011 Aug 23;6(8):e23896. doi: 10.1371/journal.pone.0023896 (PMC3160338; doi:10.1371/journal.pone.0023896)
Supplement: Table S1 — List of genes whose expression is suppressed at least 2.0 fold by AtOFP1. (DOC) [file pone.0023896.s001.doc]

**Supplementary material**

| **Supplemental Table S1.** *List of genes whose expression is suppressed at least 2.0 fold by AtOFP1.* | |
| --- | --- |
| Locus identifier | Description |
| AT3G57900 | expressed protein |
| AT5G45630 | expressed protein |
| AT5G35270 | cytochrome P-450 aromatase-related |
| AT2G07660 | gypsy-like retrotransposon |
| AT5G40050 | F-box protein |
| AT5G32490 | hypothetical protein |
| AT3G51680 | short-chain dehydrogenase/reductase |
| AT3G20160 | geranylgeranyl pyrophosphate synthase |
| AT5G51610 | ribosomal protein L11 family protein |
| AT3G44960 | expressed protein |
| AT5G13150 | exocyst subunit EXO70 family protein |
| AT1G48640 | lysine and histidine specific transporter |
| AT1G70430 | protein kinase |
| AT3G12840 | expressed protein |
| AT2G44030 | kelch repeat-containing F-box protein |
| AT5G39110 | germin-like protein |
| AT2G03180 | hypothetical protein |
| AT5G08510 | pentatricopeptide repeat-containing protein |
| AT1G53990 | GDSL-motif lipase/hydrolase family protein |
| AT2G01840 | non-LTR retrotransposon (LINE) |
| AT2G07687 | cytochrome c oxidase subunit 3 |
| AT5G39060 | hAT-like transposase |
| AT5G28790 | hypothetical protein |
| AT2G36800 | UDP-glucoronosyl/UDP-glucosyl transferase family protein |
| AT5G11410 | protein kinase |
| AT4G18530 | expressed protein |
| AT2G14520 | CBS domain-containing protein |
| AT1G66170 | PHD finger family protein (MMD1) |
| AT5G23840 | MD-2-related lipid recognition domain-containing protein |
| AT1G29630 | exonuclease |
| AT2G04920 | F-box family protein (FBX9) |
| AT2G15370 | xyloglucan fucosyltransferase |
| AT4G00540 | myb family transcription factor |
| AT4G16010 | copia-like retrotransposon family protein |
| AT1G79360 | transporter-related |
| AT5G05130 | SNF2 domain-containing protein |
| AT3G43660 | Nodulin |
| AT5G05760 | syntaxin 31 |
| AT1G12350 | DNA/panthothenate metabolism flavoprotein family protein |
| AT1G53790 | F-box family protein |
| AT1G66800 | putative alcohol dehydrogenase |
| AT1G80960 | F-box protein-related |
| AT2G04420 | expressed protein |
| AT1G02420 | pentatricopeptide (PPR) repeat-containing protein |
| AT2G32930 | zinc finger (CCCH-type) family protein |
| AT3G44070 | expressed protein |
| AT2G04070 | MATE efflux family protein |
| AT3G02930 | expressed protein |
| AT1G50310 | monosaccharide transporter (STP9) |
| AT5G13080 | WRKY family transcription factor |
| AT4G18220 | purine permease family protein |
| AT1G49245 | expressed protein |
| AT2G28270 | DC1 domain-containing protein |
| AT2G31130 | expressed protein |
| AT1G52830 | auxin-responsive protein IAA6 |
| AT1G15790 | expressed protein |
| AT4G25800 | calmodulin-binding protein |
| AT3G46710 | disease resistance protein (CC-NBS-LRR class) |
| AT1G33730 | cytochrome P450 |
| AT1G17630 | pentatricopeptide (PPR) repeat-containing protein |
| AT4G15360 | cytochrome P450 family protein |
| AT2G43600 | glycoside hydrolase family 19 protein |
| AT3G09960 | calcineurin-like phosphoesterase family protein |
| AT3G32260 | hypothetical protein |
| AT5G54230 | myb family transcription factor (MYB49) |
| AT1G20070 | expressed protein |
| AT4G14060 | major latex protein-related |
| AT2G37320 | pentatricopeptide (PPR) repeat-containing protein |
| AT5G38310 | expressed protein |
| AT1G69240 | hydrolase, alpha/beta fold family protein |
| AT4G13390 | proline-rich extensin-like family protein |
| AT3G18310 | expressed protein |
| AT5G37550 | expressed protein |
| AT3G59930 | defensin-like (DEFL) family protein |
| AT4G39190 | expressed protein |
| AT5G56520 | expressed protein |
| AT2G28260 | cyclic nucleotide-regulated ion channel |
| AT1G67980 | caffeoyl-CoA 3-O-methyltransferase |
| AT2G30840 | 2-oxoglutarate-dependent dioxygenase |
| AT1G30220 | sugar transporter family protein |
| AT1G70450 | protein kinase family protein |
| AT5G17980 | C2 domain-containing protein |
| AT1G16310 | cation efflux family protein |
| AT1G16590 | mitotic spindle checkpoint protein |
| AT4G23493 | expressed protein |
| AT5G01790 | expressed protein |
| AT5G62520 | a protein with similarity to RCD1 but without the WWE domain |
| AT1G05320 | myosin-related |
| AT4G26880 | stigma-specific Stig1 family protein |
| AT1G26840 | putative origin recognition complex subunit 6 containing protein |
| AT4G24000 | cellulose synthase family protein |
| AT1G06620 | 2-oxoglutarate-dependent dioxygenase |
| AT3G14200 | DNAJ heat shock N-terminal domain-containing protein |
| AT3G45070 | sulfotransferase family protein |
| AT3G44400 | disease resistance protein (TIR-NBS-LRR class) |
| AT2G26040 | Bet v I allergen family protein |
| AT2G07718 | putative cytochrome b |
| AT5G37500 | guard cell outward rectifying K^+^ channel (GORK) |
| AT4G33040 | glutaredoxin family protein |
| AT3G20100 | cytochrome P450 family protein |
| AT3G24840 | SEC14 cytosolic factor |
| AT1G55390 | DC1 domain-containing protein |
| AT2G01660 | 33 kDa secretory protein-related |
| AT2G13910 | putative CHP-rich zinc finger protein |
| AT4G11210 | disease resistance-responsive family protein |
| AT4G01480 | inorganic pyrophosphatase |
| AT2G26810 | expressed protein |
| AT1G21100 | O-methyltransferase |
| AT5G57950 | 26S proteasome regulatory subunit |
| AT5G44980 | F-box family protein |
| AT4G28850 | xyloglucan:xyloglucosyl transferase |
| AT3G04020 | expressed protein |
| AT4G23680 | major latex protein-related |
| AT5G66960 | prolyl oligopeptidase family protein |
| AT5G14440 | SURF2 family protein |
| AT5G67230 | glycosyl transferase family 43 protein |
| AT1G60960 | metal transporter IRT3 |
| AT1G74300 | esterase/lipase/thioesterase family protein |
| AT5G45090 | lectin-related |
| AT3G12540 | putative ternary complex factor |
| AT2G37730 | fringe-related protein |
| AT2G39680 | Trans-acting siRNA primary transcript |
| AT1G78950 | beta-amyrin synthase |
| AT2G21800 | expressed protein |
| AT1G04160 | myosin family protein |
| AT5G64510 | expressed protein |
| AT5G12220 | las1-like family protein |
| AT4G23490 | fringe-related protein |
| AT2G20520 | fasciclin-like arabinogalactan-protein (FLA6) |
